# Toward More Accurate Preoperative Diagnosis of Vein Wall Invasion in Renal Cell Carcinoma Using Computer Vision

**DOI:** 10.1093/oncolo/oyad281

**Published:** 2023-10-11

**Authors:** Valisha Shah, William Lotter

**Affiliations:** Lank Center for Genitourinary Oncology, Dana-Farber Cancer Institute, Boston, MA, USA; Department of Data Science, Dana-Farber Cancer Institute, Boston, MA, USA; Department of Pathology, Brigham and Women’s Hospital, Boston, MA, USA; Department of Pathology, Harvard Medical School, Boston, MA, USA

## Abstract

This commentary remarks on a recently published article by Zhao et al, which presents a CT radiomics model for the diagnosis of vein wall invasion in patients with renal cell carcinoma and venous tumor thrombus.

Renal cell carcinoma (RCC) is associated with an approximate 4%-10% rate of venous invasion,^1^ where this level of invasion is a significant factor in patient prognosis and surgical strategy. Deep invasive tumor thrombus (DITT) is particularly challenging to treat and requires complex surgical procedures.^2^ Toward improved preoperative diagnosis of DITT, Zhao et al^3^ present a radiomics approach to predict the presence of DITT from a patient’s preoperative CT scan. This study points to the promise of using computer vision methods to more accurately diagnose DITT, which can ultimately inform surgical planning and patient management.

Radiomics is a field of computer vision that consists of extracting quantitative features from imaging and subsequently training a machine learning classifier to predict clinical factors of interest using these quantitative features.^4^ In their study, Zhao et al develop and assess a radiomics approach using a cohort of 169 patients (111 with DITT and 58 classified with noninvasive tumor thrombus (NITT)) who were admitted to the Peking University Third Hospital. Data were split in a temporal fashion with patients who underwent surgery before September 2019 relegated to the training set (130 patients, 88 with DITT) with the remaining 39 patients assigned to the testing set (39 patients, 23 with DITT). Their modeling strategy consisted of feature extraction using the popular PyRadiomics library,^5^ followed by an elastic net regression for feature selection, and finally an extra trees classifier to predict DITT presence. This approach demonstrated promising performance with an area under the receiver operating characteristic curve (AUROC) of 0.853 (95% CI: 0.734-0.948). The authors also report subgroup analysis across several relevant clinical factors with generally consistent performance, although the small sample sizes necessarily result in large error bars.

While the presented radiomics approach demonstrates promising performance, important future efforts are necessary to realize robust clinical utility. One particularly important direction is external validation of the algorithm to assess how well the performance generalizes across clinical sites and populations. An additional common challenge in machine learning is the notion of interpretability, where there is a need for understanding the features used by the model and connecting these features to biologically meaningful concepts. Zhao et al include a thorough description of the top predictive radiomics features but connecting these often-abstract features to biological knowledge that can be used by clinicians is a challenging task. Another important future direction is the collection of larger training sets that can facilitate higher accuracy and additional modeling strategies, such as deep learning.

DITT is a challenging complication of RCC, for which approaches are needed to predict its presence more accurately on CT scans. Such approaches can ultimately guide surgical planning and patient management. Zhao et al present a radiomics model with these goals in mind that demonstrates promising performance on an internal cohort. Future validation and interpretability efforts can help bring this technology to its clinical fruition.
